# Effect of non-ionizing reaction rate (assumed to be controllable) on the plasma generation mechanism and communication around RAMC vehicle during atmospheric reentry

**DOI:** 10.1038/s41598-021-99584-3

**Published:** 2021-10-08

**Authors:** Wenchong Ouyang, Qi Liu, Zheng Zhang, Tao Jin, Zhengwei Wu

**Affiliations:** 1 School of Nuclear Science and Technology, University of Science and Technology of China, Hefei, 230026 People’s Republic of China; 2 CAS Key Laboratory of Geospace Environment, University of Science and Technology of China, Hefei, 230026 People’s Republic of China; 3 School of Aerospace Science and Technology, Xidian University, Xi’an, 710071 China; 4 The Key Laboratory of Information and Structure Efficiency in Extreme Environment, Ministry of Education of China, Xidian University, Xi’an, 710071 People’s Republic of China

**Keywords:** Engineering, Physics

## Abstract

Radio frequency (RF) blackout occurs during radio attenuation measurement C (RAMC) vehicle reentry due to the attenuation effect of the plasma sheath on the communication signal. In recent years, the mitigation mechanism of chemical reaction for RF blackout problem has gradually been studied numerically and experimentally. However, the effect of non-ionization reaction rate has been ignored because it does not directly involve the generation of electrons. In the present study, the influence of non-ionizing reaction rate on the plasma generation mechanism and EM wave attenuation was numerically solved by the plasma flow and multilayer transmission model. According to the simulation results, only the reaction rate of $NO \rightleftharpoons N + O$ has a significant effect on the electron number density in all non-ionizing reactions, and the degree of influence is less than the ionization reaction rate. The EM wave attenuation decreases with the decrease of the reaction rate of $NO \rightleftharpoons N + O$. When the reaction rate is reduced by 25 times, the maximum attenuation of electromagnetic wave can be reduced by 12 dB. Finally, a potential scheme by reducing the reaction rate of $NO \rightleftharpoons N + O$ was proposed to mitigate the RF blackout problem.

## Introduction

There is a major demand to maintain normal communication with the ground station during vehicle reentry mission. The National Aeronautics and Space Administration (NASA) developed the radio attenuation measurement C project to obtain flight data required for plasma flow and electromagnetic wave propagation models, which is to make more accurate prediction for vehicle communication during atmospheric reentry. Currently, low-cost communication methods such as electromagnetic waves (EM) can cause RF blackout during vehicle reentry. Because the speed of RAMC vehicle is much faster than 5 Mach during reentry from a low Earth orbit, the violent friction between the vehicle and air will excite the vibration of air molecules and cause chemical reactions such as dissociation and ionization. These charged free electrons, ions and neutral particles coat the surface of the vehicle, and the plasma sheath was formed. Dense plasma sheath has significant reflection and absorption effects on electromagnetic waves, and a radio frequency blackout occurs.

In order to solve the blackout problem, many models have been developed to analyze the propagation of plane waves through the reentry plasma sheath^1,2^, and many methods including shape optimization^3,4^, applied magnetic field^5,6^ and terahertz technology^7^ have been proposed. In recent years, chemical reactions under hypersonic flow have gotten widespread attention and are gradually being considered as a means to solve the blackout problem^8–10^. The chemical reactions currently used for the simulation of plasma flow around reentry vehicle almost come from the models of park^11,12^ and Gupta^13^. These models are suitable for a wide temperature range, but are determined based on experimental results measured at a specific temperature. Therefore, it is meaningful to consider the uncertainty of chemical reactions in plasma flow simulation. Jung et al. first numerically analyzed the impact of the uncertainty of chemical reaction on the plasma generation and RF blackout prediction, and found that the associative ionization reaction of nitrogen has the greatest effect^14^. The results of further quantitative analysis by Ouyang et al. showed that the reaction rate of $N + O \rightleftharpoons NO^{ + } + e$ is reduced by 25 times, and the electromagnetic wave attenuation is reduced by about 30 dB^15^. Takahashi et al. considered the effects of entire surface catalysis on the RF blackout, and the results showed that the catalysis of ionization reactions can effectively reduce signal attenuation^16^.

Obviously, these studies^14–16^ on the chemical reaction rate for blackout problem are based on the assumption that it is controllable. However, recent experimental demonstration of mitigating reentry blackout via surface catalysis effects revealed the possibility and significance of chemical reaction rates for blackout problem^17^. The ionization reaction has been focused on by many researchers because it directly involves the generation of electrons, but the non-ionization reaction was ignored. Although there is no generation of electrons in non-ionization reactions, reactions such as $NO \rightleftharpoons N + O$ and $N_{2} \rightleftharpoons N + N$ directly affect the formation of *N* and *O*, thereby affecting ionization reactions such as $N + O \rightleftharpoons NO^{ + } + e$. Therefore, the influence of the non-ionizing reaction rate on the plasma generation mechanism and EM wave communication around the vehicle was studied in this paper. It is worth noting that the non-ionization reaction rate is assumed to be controllable due to the numerical simulation of research method.

In the present paper, the influence of the non-ionizing reaction rate on the electron number density and collision frequency was first solved by the finite volume method. Based on the results, the effect of non-ionizing reaction rate on the attenuation of electromagnetic waves around the vehicle was solved by multilayer transmission model. Finally, plasma generation mechanism and a potential approach about non-ionization reaction rate for blackout problem were discussed.

## Methods and data validation

### Vehicle description and calculation conditions

**Figure 1:**
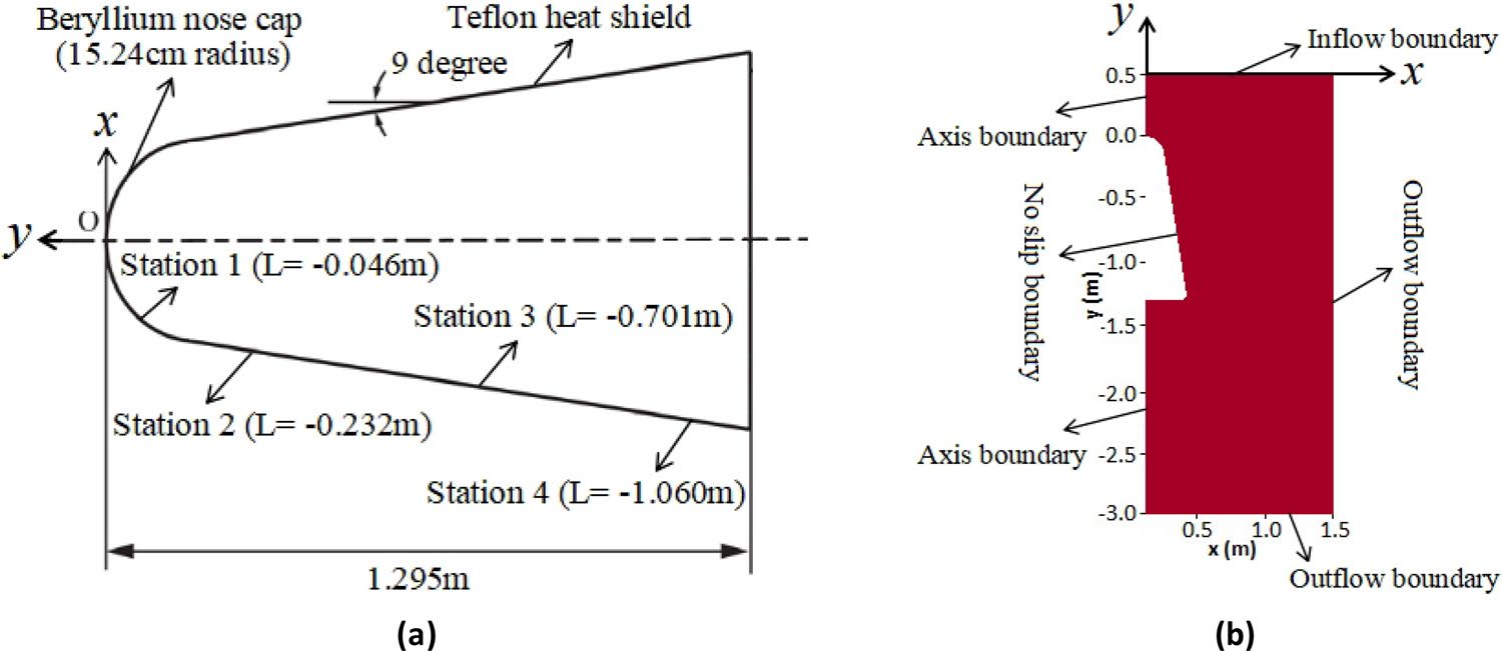
Shape configuration (**a**) and calculation oundary condition (**b**) of RAMC vehicle.

The RAMC vehicle of the NASA was selected as the research object in this paper. The vehicle was launched from NASA's Wallops station, and the reentry process took place in Bermuda where tracking and telemetry stations were set up. The mission of The RAMC vehicle is to measure the plasma related data of nosecap and tail, so as to further deepen the understanding and verify the plasma theoretical model^18^. The shape of the RAMC vehicle is shown in Fig. 1a. This is a typical blunt-body structure, the nosecap radius and half-angle of the vehicle are 15.24 cm and 9°, respectively. Microwave antennas are installed in Station 1–4, as detailed in Fig. 1a. L = − 0.046 m indicates that the distance between the antenna position and the top of the vehicle is 0.046 m. It should be noted that the ablating teflon was applied to the surface of the vehicle for thermal protection, and the ablation product has a certain effect on the electron density.

RAMC vehicle is axisymmetric, so only half of the vehicle is used for numerical simulation. The calculation area and boundary condition for plasma simulation are shown in Fig. 1b. The setting of boundary conditions is mainly divided into the following four parts: the axis boundary, no slip boundary, inflow boundary and outflow boundary.

The initial conditions of free flow reported by Grantham et al.^18,19^ were applied to the inflow boundary, as show in Table 1. All flow variables at the outflow boundary were set to no gradient.

**Table 1:** Free stream parameters for different reentry heights.

Altitude, km	Velocity, m/s	Temperature, K	Density, kg/m^3^	Mach number
20	3290	211.6	8.804 × 10^−2^	11.3
30	6550	231.2	1.801 × 10^−2^	21.5
40	7380	253.2	4.360 × 10^−3^	23.2
50	7620	266.2	1.150 × 10^−3^	23.3
61	7650	244.3	2.816 × 10^−4^	24.4
70	7650	210.9	8.830 × 10^−5^	26.3
80	7650	195.8	1.850 × 10^−5^	27.3

### Numerical method

**Figure 2:**
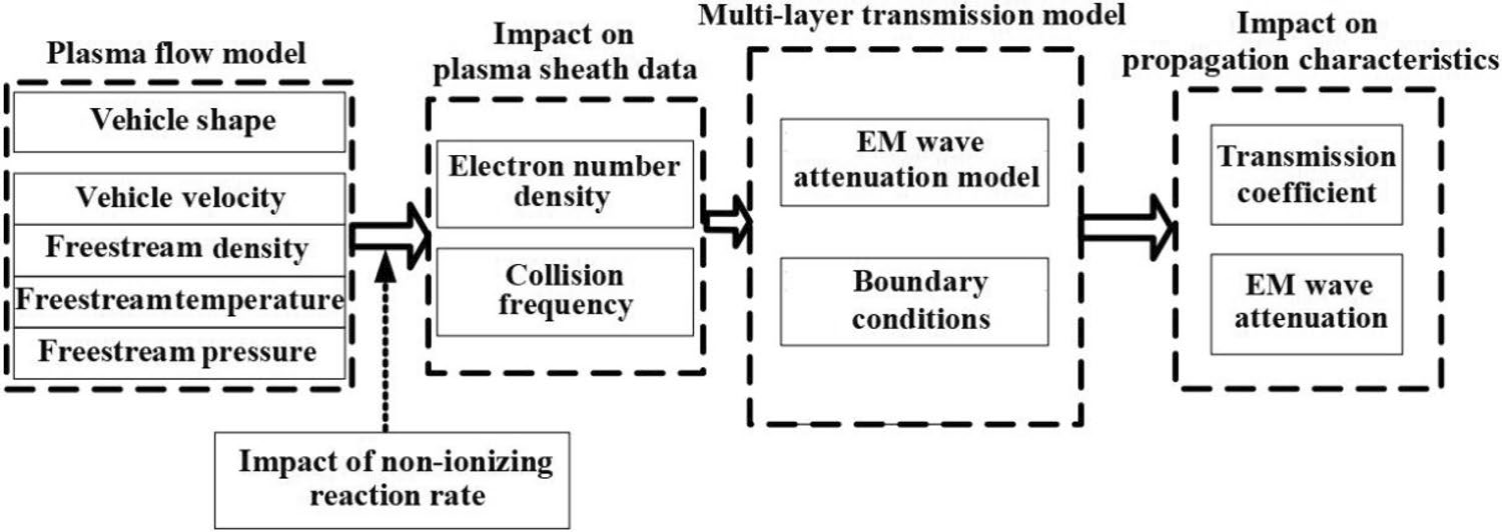
Combined simulation of plasma flow and multi-layer transmission model.

The combined simulation model of plasma flow and electromagnetic wave propagation is mainly divided into two parts, as shown in Fig. 2. First, based on the initial conditions of the free stream and the configuration of vehicle, the plasma model is applied to solve the effect of non-ionization reaction rate on the electron number density and electron collision frequency. Second, the data of electron number density and electron collision frequency are used as the input of the multi-layer transmission model to solve the influence of the non-ionization reaction rate on the attenuation of electromagnetic waves.

### Plasma flow model

The plasma flow model^15^ consists of the Navier–Stokes equations and the chemical reaction model. The Navier–Stokes equations includes the conservation of species mass, momentum, and total energy.

1 $$ \frac{{\partial\uprho }}{\partial t} + \nabla \cdot \left( {\rho \vec{u}} \right) = 0 $$

2 $$ \frac{{\partial \left( {\rho \vec{u}} \right)}}{\partial t} + \nabla \cdot \left( {\rho \vec{u}\vec{u} + p{\vec{\vec{I}}} } \right) = \nabla \cdot {\vec{\vec{\tau }}} $$

3 $$ \frac{\partial E}{{\partial t}} + \nabla \cdot \left( {\vec{u}\left( {E + p} \right)} \right) = \nabla \cdot \left( {{\vec{\vec{\tau }}} \cdot \vec{u}} \right) + \nabla \cdot \left( {k_{T} \nabla T} \right) + \nabla \cdot \left( {\rho \mathop \sum \limits_{i} H_{i} D_{i} \nabla w_{i} } \right) $$

where ρ is the mass density, $$\vec{u}$$ is the velocity, $$p$$ is the pressure, $${\vec{\vec{I}}}$$ is the identity matrix, $${\vec{\vec{\tau }}}$$ is the stress tensor, $$i$$ is the index of the species. $$E$$, $$k_{T}$$, $$ T$$, $$H_{i}$$, $$D_{i}$$, $$w_{i}$$ are the total energy, thermal conductivity, temperature, enthalpy of formation, diffusion coefficient, mass fraction of the gas component, respectively.

The 7 component and 18 chemical reaction model was used to simulate the high temperature area reaction during atmospheric reentry. The relevant reaction parameters are obtained from the report of Park et al.^11–13^. The forward reaction rate is calculated according to the following equation:

4 $$ k_{f} \left( {T_{r} } \right) = A_{c} \left( {\frac{{T_{r} }}{298}} \right)^{{n^{\prime}}} exp\left( { - \frac{{E_{a} }}{{T_{r} }}} \right) $$

The equilibrium rate is calculated as:

5 $$ k_{e} \left( {T_{r} } \right) = B_{c} exp\left( {\mathop \sum \limits_{i = 1}^{5} c_{i} \left( {\frac{10000}{{T_{r} }}} \right)^{i} } \right) $$

The parameters $c_{1}$ through $c_{5}$ for the reactions are presented in Table 1. The backward reaction rate is expressed as the ratio of the forward reaction rate to the equilibrium rate.

6 $$ k_{b} \left( {T_{r} } \right) = \frac{{ k_{f} \left( {T_{r} } \right)}}{{ k_{e} \left( {T_{r} } \right)}} $$

where $k_{f}$ is the forward reaction rate, $T_{r}$ is the reaction temperature, $A_{C}$ is the forward reaction rate coefficient, $n^{\prime}$ is the forward reaction rate exponent, $E_{a}$ is the characteristic reaction temperature, $k_{e}$ is the equilibrium reaction rate, $B_{C}$ and $c_{i}$ are the backward reaction rate coefficient, $k_{b}$ is the backward reaction rate.

The chemical reaction will cause the density of each species to change, but it conforms to the law of conservation.

7 $$ \frac{{\partial {\text{n}}_{i} }}{\partial t} + \nabla \cdot \left( {\vec{u}{\text{n}}_{i} } \right) = {\text{s}}_{i} $$

where ${\text{s}}_{i}$ is the change rate of number density of each species.

The specific chemical reactions and reaction coefficients are shown in Table 2^11–13,15^. The generation of electrons comes from the ionization reaction $N + O \rightleftharpoons NO^{ + } + e$, but the formation of N and O in the non-ionization reaction will indirectly affect the ionization reaction. The non-ionization reactions in this reaction model are mainly $N_{2} \rightleftharpoons N + N$ (reaction 1–5), $O_{2} \rightleftharpoons O + O$ (reaction 5–10) and $NO \rightleftharpoons N + O$ (reactions 11–15).

**Table 2:** Chemical reactions and reaction coefficients.

r	Chemical reaction	${\text{A}}_{{\text{c}}}$	n′	E_a_	${\text{B}}_{{\text{c}}}$	m′	c_1_	c_2_	c_3_	c_4_	c_5_
1	${\text{N}}_{2} + {\text{N}}_{2} \rightleftharpoons {\text{N}} + {\text{N}} + {\text{N}}_{2}$	f_1_ × 6.76 × 10^−13^	− 1.6	9.41 × 10^5^	1.66 × 10^−27^	5	3.898	− 12.611	0.683	− 0.118	0.006
2	${\text{N}}_{2} + {\text{O}}_{2} \rightleftharpoons {\text{N}} + {\text{N}} + {\text{O}}_{2}$	f_1_ × 6.76 × 10^−13^	− 1.6	9.41 × 10^5^	1.66 × 10^−27^	5	3.898	− 12.611	0.683	− 0.118	0.006
3	${\text{N}}_{2} + {\text{NO}} \rightleftharpoons {\text{N}} + {\text{N}} + {\text{NO}}$	f_1_ × 6.76 × 10^−13^	− 1.6	9.41 × 10^5^	1.66 × 10^−27^	5	3.898	− 12.611	0.683	− 0.118	0.006
4	${\text{N}}_{2} + {\text{N}} \rightleftharpoons {\text{N}} + {\text{N}} + {\text{N}}$	f_1_ × 2.01 × 10^−12^	− 1.6	9.41 × 10^5^	1.66 × 10^−27^	5	3.898	− 12.611	0.683	− 0.118	0.006
5	${\text{N}}_{2} + {\text{O}} \rightleftharpoons {\text{N}} + {\text{N}} + {\text{O}}$	f_1_ × 2.01 × 10^−12^	− 1.6	9.41 × 10^5^	1.66 × 10^−27^	5	3.898	− 12.611	0.683	− 0.118	0.006
6	${\text{O}}_{2} + {\text{N}}_{2} \rightleftharpoons {\text{O}} + {\text{O}} + {\text{N}}_{2}$	f_2_ × 1.53 × 10^−13^	− 1.0	4.95 × 10^5^	1.66 × 10^−27^	5	1.335	− 4.127	− 0.616	0.093	− 0.005
7	${\text{O}}_{2} + {\text{O}}_{2} \rightleftharpoons {\text{O}} + {\text{O}} + {\text{O}}_{2}$	f_2_ × 1.53 × 10^−13^	− 1.0	4.95 × 10^5^	1.66 × 10^−27^	5	1.335	− 4.127	− 0.616	0.093	− 0.005
8	${\text{O}}_{2} + {\text{NO}} \rightleftharpoons {\text{O}} + {\text{O}} + {\text{NO}}$	f_2_ × 1.53 × 10^−13^	− 1.0	4.95 × 10^5^	1.66 × 10^−27^	5	1.335	− 4.127	− 0.616	0.093	− 0.005
9	${\text{O}}_{2} + {\text{N}} \rightleftharpoons {\text{O}} + {\text{O}} + {\text{N}}$	f_2_ × 4.60 × 10^−10^	− 1.0	4.95 × 10^5^	1.66 × 10^−27^	5	1.335	− 4.127	− 0.616	0.093	− 0.005
10	${\text{O}}_{2} + {\text{O}} \rightleftharpoons {\text{O}} + {\text{O}} + {\text{O}}$	f_2_ × 4.60 × 10^−10^	− 1.0	4.95 × 10^5^	1.66 × 10^−27^	5	1.335	− 4.127	− 0.616	0.093	− 0.005
11	${\text{NO}} + {\text{N}}_{2} \rightleftharpoons {\text{N}} + {\text{O}} + {\text{N}}_{2}$	f_3_ × 3.82 × 10^−13^	0.0	6.28 × 10^5^	1.66 × 10^−27^	5	1.549	− 7.784	0.228	− 0.043	0.002
12	${\text{NO}} + {\text{O}}_{2} \rightleftharpoons {\text{N}} + {\text{O}} + {\text{O}}_{2}$	f_3_ × 3.82 × 10^−13^	0.0	6.28 × 10^5^	1.66 × 10^−27^	5	1.549	− 7.784	0.228	− 0.043	0.002
13	${\text{NO}} + {\text{NO}} \rightleftharpoons {\text{N}} + {\text{O}} + {\text{NO}}$	f_3_ × 3.82 × 10^−13^	0.0	6.28 × 10^5^	1.66 × 10^−27^	5	1.549	− 7.784	0.228	− 0.043	0.002
14	${\text{NO}} + {\text{N}} \rightleftharpoons {\text{N}} + {\text{O}} + {\text{N}}$	f_3_ × 4.42 × 10^−14^	− 0.5	6.28 × 10^5^	1.66 × 10^−27^	5	1.549	− 7.784	0.228	− 0.043	0.002
15	${\text{NO}} + {\text{O}} \rightleftharpoons {\text{N}} + {\text{O}} + {\text{O}}$	f_3_ × 4.42 × 10^−14^	− 0.5	6.28 × 10^5^	1.66 × 10^−27^	5	1.549	− 7.784	0.228	− 0.043	0.002
16	${\text{N}}_{2} + {\text{O}} \rightleftharpoons {\text{NO}} + {\text{N}}$	2.99 × 10^−17^	− 0.1	3.13 × 10^5^	1.0	5	2.349	− 4.828	0.455	− 0.075	0.004
17	${\text{NO}} + {\text{O}} \rightleftharpoons {\text{O}}_{2} + {\text{N}}$	5.58 × 10^−19^	1.29	1.60 × 10^5^	1.0	5	0.215	− 3.652	0.843	− 0.136	0.007
18	${\text{N}} + {\text{O}} \rightleftharpoons {\text{NO}}^{ + } + {\text{e}}$	f_4_ × 1.08 × 10^−18^	0.0	2.66 × 10^5^	1.0	5	− 6.234	− 5.536	0.494	− 0.058	0.003

The chemical reaction model is applicable to a wide temperature range, but the reaction coefficient is derived from experimental data at a certain temperature. Therefore, the non-ionization reaction rate has a certain degree of uncertainty. A parametric study of non-ionization reaction rate was performed to evaluate its impact on the electron density and communication around the vehicle. The forward reaction rate factors of $N_{2} \rightleftharpoons N + N$, $O_{2} \rightleftharpoons O + O$, $NO \rightleftharpoons N + O$ and $N + O \rightleftharpoons NO^{ + } + e$ are $f_{1}$, $f_{2}$, $f_{3}$ and $f_{4}$, respectively. The values of $f_{1}$, $f_{2}$, $f_{3}$ and $f_{4}$ are all equal to 1 under the standard reaction model, the reaction rate conditions for different non-ionizing reactions are summarized in Table 3.

**Table 3:** Factors of forward non-ionization reaction rates.

Factor	Case 1	Case 2	Case 3	Case 4	Case 5	Case 6	Case 7	Case8	Case 9
f_1_	1	0.04	10	1	1	1	1	1	1
f_2_	1	1	1	0.04	10	1	1	1	1
f_3_	1	1	1	1	1	0.04	10	1	1
f_4_	1	1	1	1	1	1	1	0.04	10

### Multi-layer transmission model

**Figure 3:**
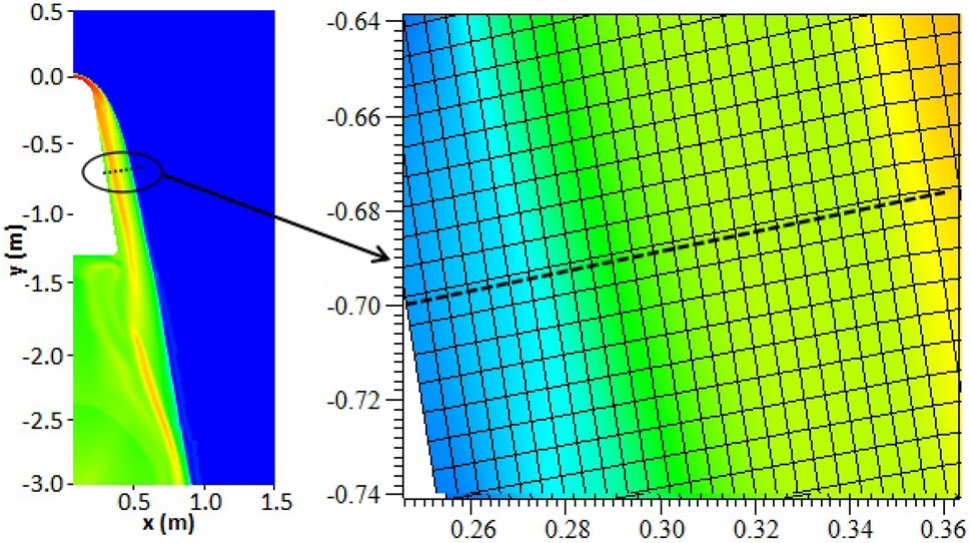
An enlarged schematic diagram of the plasma sheath near the antenna.

In the RAMC flight experiment, microwave antennas were installed at four positions on the surface of the vehicle, which are represented as Station 1–4 in Fig. 1^18^. Therefore, in the electromagnetic wave propagation simulation, the antennas are installed at a moderate position L = − 0.4 m. Figure 3 is an enlarged schematic diagram of the plasma sheath near the antenna installation position y = − 0.701 m. It can be seen from Fig. 3 that the plasma sheath is divided into m uniform thin layers, and the plasma parameters in each thin layer correspond to the grid data calculated by the plasma flow model. Therefore, the multi-layer transmission model was applied to calculate the attenuation of electromagnetic waves. The multi-layer transmission model used here is almost the same as those reported previously, and the reliability of the model has been examined^4,20,21^.

### Data validation

**Figure 4:**
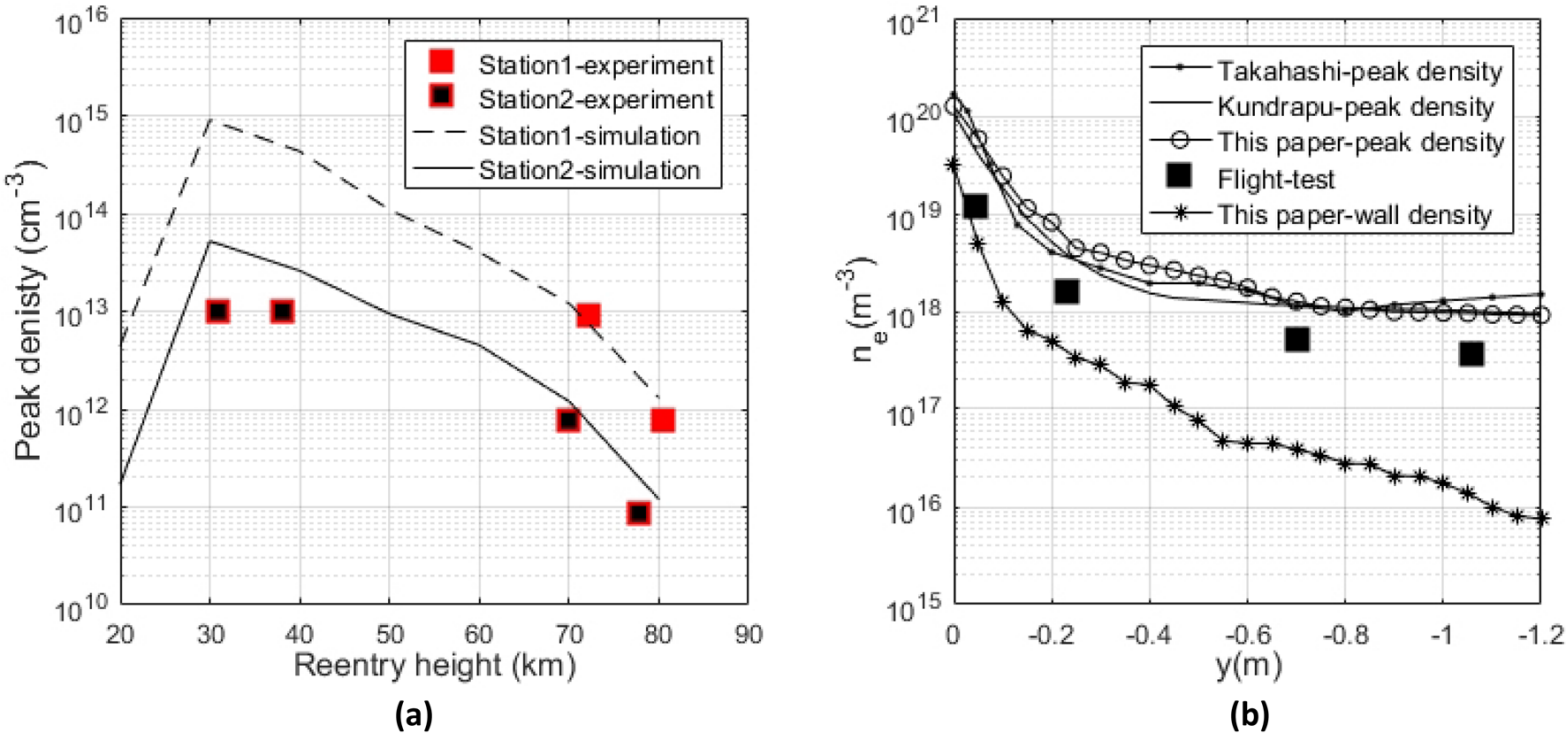
Comparison of electron number density distribution with flight test (**a**) and other researchers’ results (**b**).

Based on the above model and calculation conditions, the plasma distribution around vehicle was calculated. Figure 4a compares the electron density between the plasma flow simulation and flight test data. The electron number density of the numerical simulation is in good agreement with the measurement data of flight test. However, the electron density of the simulation result at 30 km and 40 km is slightly higher than that measured by the reflectometer. The reason is that the error generated by the electron density determined by the reflectometer at low altitude is higher than that at high altitude^18^. The error is mainly due to the collision frequency effect that reduces the clarity of reflection coefficient change at the critical density. In addition, the ablation effect on the antenna is also a factor that causes errors. The flow field distribution around the vehicle is significantly different at different reentry heights, the reentry height is set at 61 km in this paper. Figure 4b compares the electron number density distribution at 61 km with the simulation results of other researchers^22,23^ and the reflectometer measurements presented in reference^18^. Although the plasma flow and chemical reaction models in this paper are different from those in reference^22,23^, the simulation results in this paper are in good agreement with other researchers. Since the reflectometer measures the approximate average electron density of a small area, it is completely reasonable that the flight test data is between the peak density and the wall density. In addition, the wall density is lower than the peak density, the reason is that the wall temperature is lower than the boundary layer temperature. The above comparison results show the accuracy of the simulation results in this paper.

## Results and discussion

### Impact of non-ionization reaction rate on plasma flow

**Figure 5:**
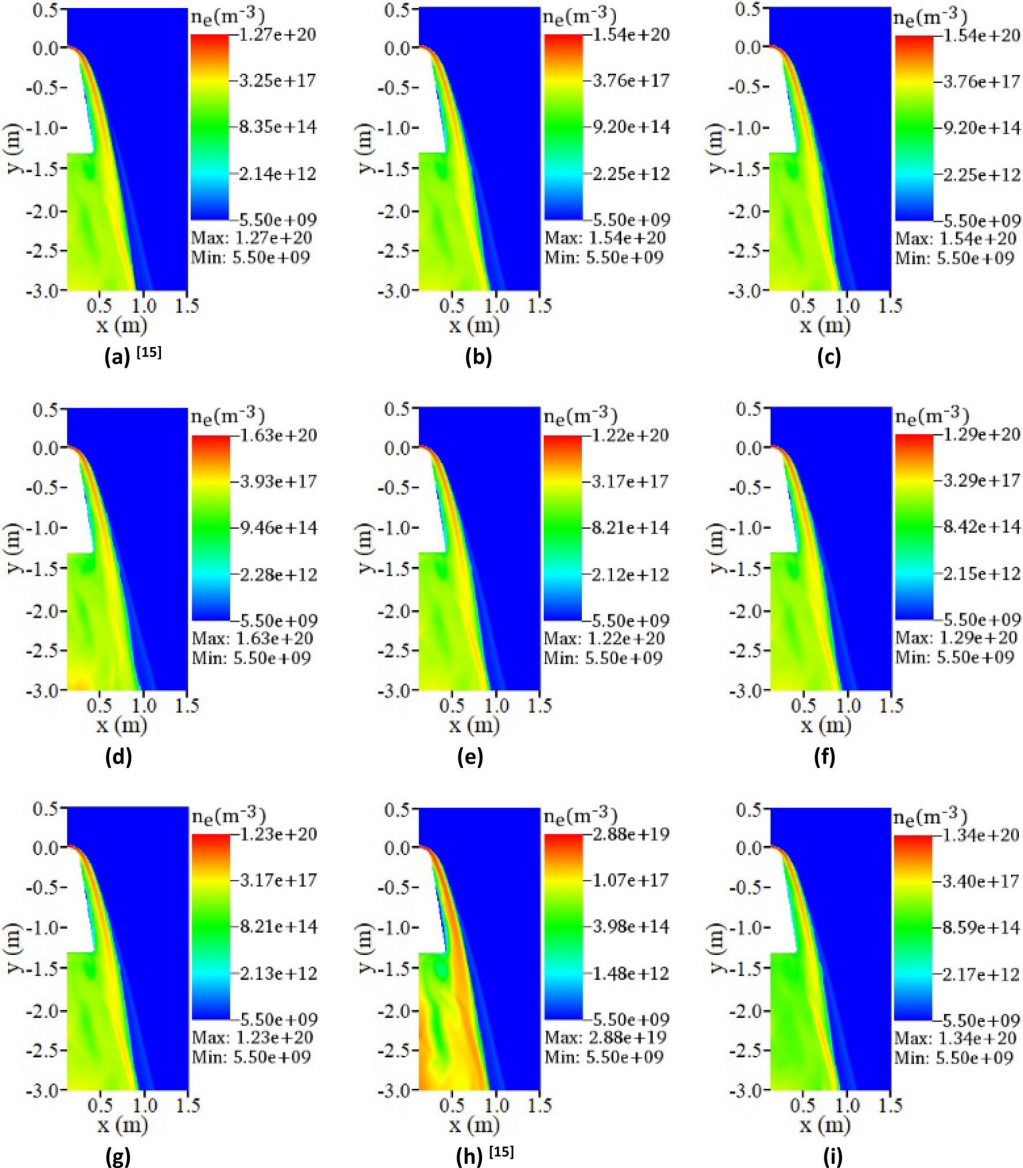
The electron number density distribution in Cases1-9 at 61 km. (**a**) case1 (f_1_ = 1, f_2_ = 1, f_3_ = 1, f_4_ = 1), (**b**) case2 (f_1_ = 0.04, f_2_ = 1, f_3_ = 1, f_4_ = 1), (**c**) case3 (f_1_ = 10, f_2_ = 1, f_3_ = 1, f_4_ = 1), (**d**) case4 (f_1_ = 1, f_2_ = 0.04, f_3_ = 1, f_4_ = 1), (**e**) case5 (f_1_ = 1, f_2_ = 10, f_3_ = 1, f_4_ = 1), (**f**) case6 (f_1_ = 1, f_2_ = 1, f_3_ = 0.04, f_4_ = 1), (**g**) case7 (f_1_ = 1, f_2_ = 1, f_3_ = 10, f_4_ = 1), (**h**) case8 (f_1_ = 1, f_2_ = 1, f_3_ = 1, f_4_ = 0.04), (**i**) case9 (f_1_ = 1, f_2_ = 1, f_3_ = 1, f_4_ = 10).

Figure 5 shows the electron density distribution around the vehicle at 61 km in cases 1–9. Among them, Fig. 5a–c show the influence of reaction rate of $N_{2} \rightleftharpoons N + N$ (case1, case2, case3) on the electron density distribution, Fig. 5a,d and e show the influence of reaction rate of $O_{2} \rightleftharpoons O + O$ (case1, case4, case5) on the electron density distribution, Fig. 5a,f and g show the influence of reaction rate of $NO \rightleftharpoons N + O$ (case1, case6, case7) on the electron density distribution, Fig. 5a,h and i show the influence of reaction rate of $N + O \rightleftharpoons NO^{ + } + e$ (case1, case8, case9) on the electron density distribution.

It is obvious from Fig. 5a–g that the non-ionizing reaction rate does not affect the formation of shock waves and plasma sheaths. The reason is that the change of the non-ionization reaction rate does not prevent the occurrence of chemical reactions. It only affects the generation of electrons by affecting the production of substances such as N and O, which makes the electron number density change. It can also be seen from Fig. 5 that the impact of the non-ionization reaction rate on the electron number density is also limited, and much smaller than the effect of ionization reaction rate. The ionization reaction $N + O \rightleftharpoons NO^{ + } + e$ directly involves the generation of electrons, and its influence on the electron density can be considered as a direct influence. Non-ionization reactions such as $N_{2} \rightleftharpoons N + N$, $O_{2} \rightleftharpoons O + O$ and $NO \rightleftharpoons N + O$ are considered to be secondary influence. Because they do not involve the generation of electrons, and affect the ionization reaction $N + O \rightleftharpoons NO^{ + } + e$ by affecting the generation of N and O, thereby further affecting the electron density. Therefore, the degree of direct influence is much higher than the degree of secondary influence, which has also been verified in the above discussion.

**Figure 6:**
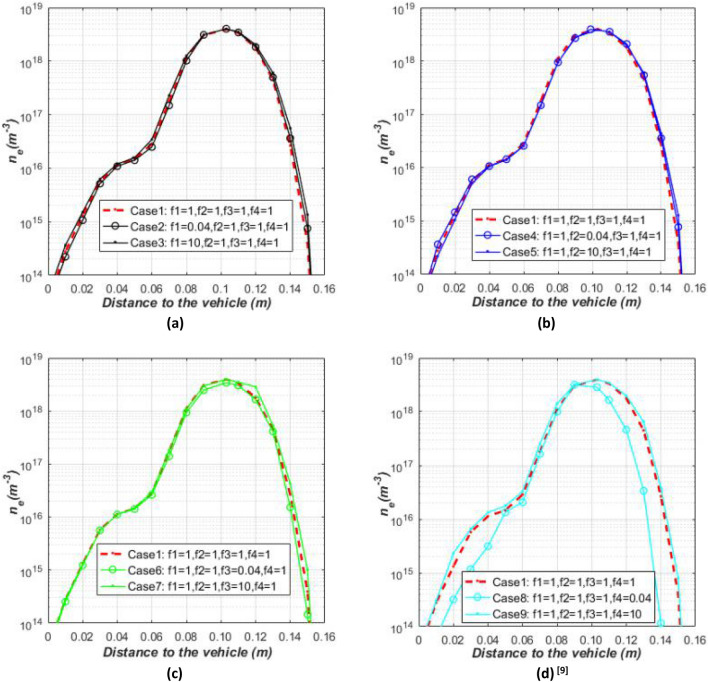
Influence of different non-ionization reaction rates on the electron number density and the comparison with the influence of ionization reaction rate. (**a**) Reaction rate of $N_{2} \rightleftharpoons N + N$, (**b**) reaction rate of $O_{2} \rightleftharpoons O + O$, (**c**) reaction rate of $NO \rightleftharpoons N + O$ and (**d**) reaction rate of $N + O \rightleftharpoons NO^{ + } + e$.

In order to compare the changes of electron number density under different non-ionization reaction rates more intuitively, the antenna is set at a moderate position L = − 0.4 m in this paper, and the electron number density in the plasma sheath around the antenna position (L = − 0.4 m) is specifically analyzed. Figure 6a–c show the effect of different non-ionization reaction rates on the electron number density distribution near the antenna. Figure 6d shows the influence of ionization reaction rate on the electron number density distribution, which has been analyzed in the previous study^15^ and is used in this paper to better compare with the effects of non-ionization reactions. It can be seen from Fig. 6 that the non-ionization reaction rate has much less influence on the electron number density than the ionization reaction rate. The reaction rate of $N_{2} \rightleftharpoons N + N$ and $O_{2} \rightleftharpoons {\text{O}} + {\text{O}}$ has almost no effect on the electron density around the antenna. The reaction rate of $NO \rightleftharpoons N + O$ and $N + O \rightleftharpoons NO^{ + } + e$^15^ is reduced by about 25 times, and the electron number density is reduced by about 30% and 5 times, respectively.

Although the decrease in the reaction rate of $N_{2} \rightleftharpoons N + N$ and $O_{2} \rightleftharpoons O + O$ reduces the formation of *N* or *O* in this reaction, the electron number density does not decrease. The reason is that a single reduction of a certain substance of *N* or *O* will promote the formation of the substance in other reactions. Take $N_{2} \rightleftharpoons N + N$ reaction as an example, reducing the reaction rate of $N_{2} \rightleftharpoons N + N$ will reduce the formation of N, the reduction of N is equivalent to the reduction of the product content for the reaction of $N_{2} + O \rightleftharpoons NO + N$ and $NO + O \rightleftharpoons O_{2} + N$, which promotes the forward reaction so that the content of *N* increases, and finally reaches an equilibrium state. For the $NO \rightleftharpoons N + O$ reaction, the decrease of the reaction rate reduces the content of N and O in the same proportion, so the content of the reactant O in $N_{2} + O \rightleftharpoons NO + N$ and $NO + O \rightleftharpoons O_{2} + N$ reaction increases in the same proportion while the content of the product N also increases. Therefore, the final reduction in N and O leads to a reduction in the electron number density in the ionization reaction of $N + O \rightleftharpoons NO^{ + } + e$, although the effect is not so significant.

**Figure 7:**
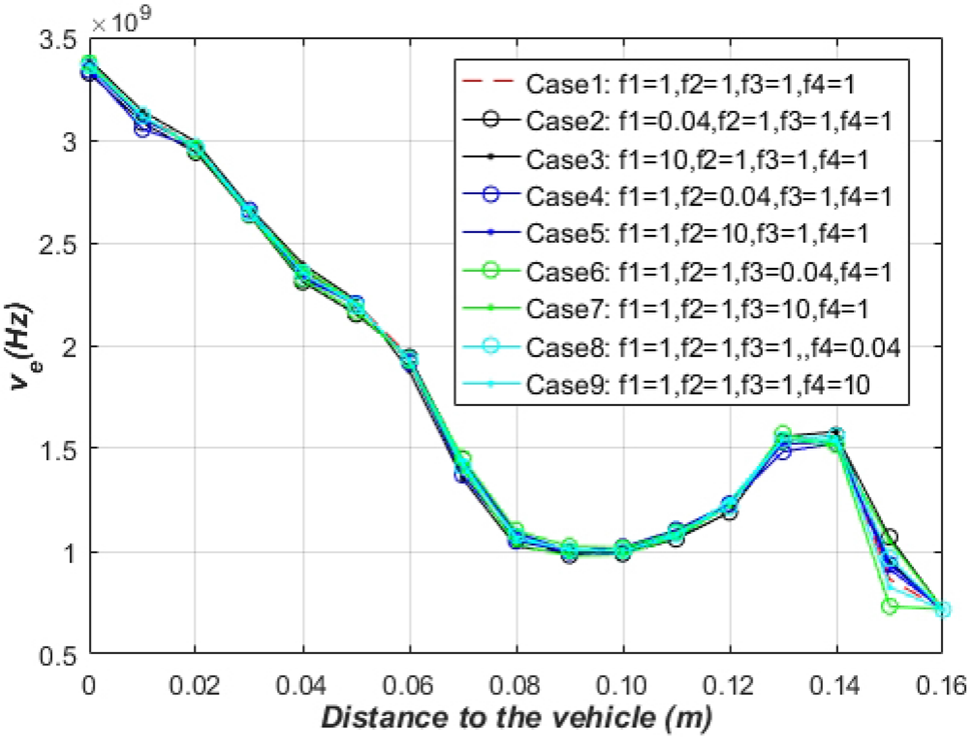
Influence of different non-ionization reaction rates and ionization reaction rates on the electron collision frequency.

Figure 7 shows the distribution of the electron collision frequency along the distance to the vehicle in cases 1–9. It is obvious that the ionization reaction rate has almost no effect on the collision frequency, which is consistent with the analysis by Ouyang et al.^15^. At the same time, the change of the non-ionizing reaction rate did not affect the electron collision frequency. The reason is that the non-ionization reaction rate has little effect on the formation of *N* and *O* substances in the non-ionization reaction, resulting in almost no effect on the pressure. Combined with Eq. (8), it can be analyzed that there is almost no effect on the frequency of electron collision.

8 $$ v_{e} = 5.814 \times 10^{12} \frac{p}{{p_{0} }}T^{{ - \frac{1}{2}}} $$

where $v_{e}$ and $p_{0}$ represent collision frequency and standard atmospheric pressure, respectively.

### Impact of non-ionization reaction rate on electromagnetic wave attenuation

Through the above analysis of the influence of the non-ionizing reaction rate on the electron number density and collision frequency, the reaction rate of $N_{2} \rightleftharpoons N + N$ and $O_{2} \rightleftharpoons O + O$ has almost no effect on the collision frequency and electron number density. Compared with them, the reaction rate in $NO \rightleftharpoons N + O$ has a more significant influence on the electron number density. Therefore, the influence of the reaction rate on the attenuation of electromagnetic wave in the $NO \rightleftharpoons N + O$ non-ionization reaction was mainly analyzed and compared with the influence of the ionization reaction rate in this section.

**Figure 8:**
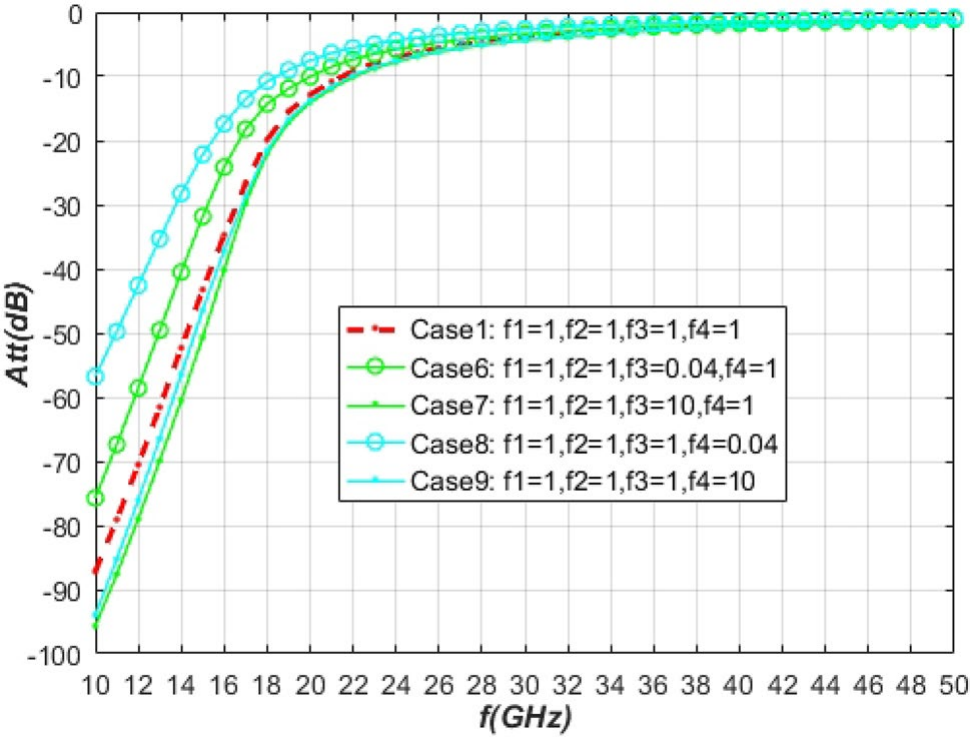
Attenuation of electromagnetic waves near the antenna under different ionization and non-ionization reaction rates.

Figure 8 shows the influence of different non-ionization reaction rates and ionization reaction rates on the attenuation of electromagnetic waves. Comparing the electromagnetic wave attenuation under case1 and case8, the ionization reaction rate is reduced by 25 times, and the electromagnetic wave attenuation is reduced by about 30 dB. This is consistent with the conclusion of Ouyang et al.^15^, and further verifies the accuracy of the electromagnetic wave calculation model in this paper.

Comparing the electromagnetic wave attenuation in Case6 and Case8 with Case1, the electromagnetic wave attenuation decreases when the reaction rate of non-ionization reaction $NO \rightleftharpoons N + O$ or ionization reaction $N + O \rightleftharpoons NO^{ + } + e$ decreases, and the influence of non-ionization reaction is lower than that of ionization reaction. The reason is that reducing the $NO \rightleftharpoons N + O$ and $N + O \rightleftharpoons NO^{ + } + e$ reaction rates both reduces the electron number density near the antenna, and the reduction of the $N + O \rightleftharpoons NO^{ + } + e$ reaction rate has a more significant effect on reducing the electron number density. If the frequency of the wave used for communication is 10 GHz, the attenuation of the electromagnetic wave will be reduced by about 12 dB when the reaction rate of the non-ionization reaction $NO \rightleftharpoons N + O$ is reduced by 25 times. The general standard for maintaining normal communication is that the attenuation is less than 5 dB, and the corresponding communication frequencies in Case 1, Case 6, and Case 8 are 28 GHz, 25 GHz, and 22 GHz, respectively.

Based on the above analysis, in the non-ionization reaction, only the reaction rate of $NO \rightleftharpoons N + O$ has a significant impact on the electron density and electromagnetic wave attenuation around the vehicle. Although the effect of the $NO \rightleftharpoons N + O$ reaction rate is less than the ionization reaction rate $N + O \rightleftharpoons NO^{ + } + e$, reducing the reaction rate of $NO \rightleftharpoons N + O$ can still effectively reduce the attenuation of electromagnetic waves.

## Conclusion

This paper mainly studied the effect of non-ionization reaction rate on the generation mechanism and electromagnetic wave attenuation around the vehicle, and compares it with the effect of the ionization reaction rate. Our results showed that non-ionization reaction has less influence on electron density and electromagnetic wave communication than that of ionization reaction. The reason is that the ionization reaction is a direct effect, while the non-ionization reaction is a secondary effect. The ionization reaction directly involves the production of electrons, which has a greater impact on the electron density. Electron density, as the main factor affecting the propagation of electromagnetic waves, will further affect the communication around RAMC vehicle. In all non-ionization reactions, only the reaction rate of $NO \rightleftharpoons N + O$ has a significant impact on the electron density and electromagnetic wave attenuation around the vehicle in the non-ionization reaction. It may be that the reduction of the reaction rate of $NO \rightleftharpoons N + O$ will reduce the formation concentration of *N* and *O* at the same time, so that the forward reaction rate of $N + O \rightleftharpoons NO^{ + } + e$ will decrease, thereby making electrons density decreases. Non-ionization reactions such as $N_{2} \rightleftharpoons N + N$ and $O_{2} \rightleftharpoons O + O$ only change the generation of $N$ or ${\text{O}}$, so the impact degree is not as great as $NO \rightleftharpoons N + O$*.* Although the effect of the $NO \rightleftharpoons N + O$ reaction rate is less than the ionization reaction rate ${\text{N}} + {\text{O}} \rightleftharpoons NO^{ + } + {\text{e}}$, the signal attenuation can still be reduced by about 12 dB when the ionization rate is reduced by 25 times.

Based on the above numerical research results, a potential approach about reducing non-ionization reaction rate was proposed to mitigate the blackout problem. The chemical reactions under hypersonic flow is a very complicated process, and there are still many problems that need to be improved. Meanwhile, the experiment of the characteristic chemical reaction rate modulation still expected to be carried out step by step.
